# Structural and Regulatory Elements of HCV NS5B Polymerase – β-Loop and C-Terminal Tail – Are Required for Activity of Allosteric Thumb Site II Inhibitors

**DOI:** 10.1371/journal.pone.0084808

**Published:** 2014-01-09

**Authors:** Sarah E. Boyce, Neeraj Tirunagari, Anita Niedziela-Majka, Jason Perry, Melanie Wong, Elaine Kan, Leanna Lagpacan, Ona Barauskas, Magdeleine Hung, Martijn Fenaux, Todd Appleby, William J. Watkins, Uli Schmitz, Roman Sakowicz

**Affiliations:** Gilead Sciences Inc., Foster City, California, United States of America; Institute of Enzymology of the Hungarian Academy of Science, Hungary

## Abstract

Elucidation of the mechanism of action of the HCV NS5B polymerase thumb site II inhibitors has presented a challenge. Current opinion holds that these allosteric inhibitors stabilize the closed, inactive enzyme conformation, but how this inhibition is accomplished mechanistically is not well understood. Here, using a panel of NS5B proteins with mutations in key regulatory motifs of NS5B – the C-terminal tail and β-loop – in conjunction with a diverse set of NS5B allosteric inhibitors, we show that thumb site II inhibitors possess a distinct mechanism of action. A combination of enzyme activity studies and direct binding assays reveals that these inhibitors require both regulatory elements to maintain the polymerase inhibitory activity. Removal of either element has little impact on the binding affinity of thumb site II inhibitors, but significantly reduces their potency. NS5B in complex with a thumb site II inhibitor displays a characteristic melting profile that suggests stabilization not only of the thumb domain but also the whole polymerase. Successive truncations of the C-terminal tail and/or removal of the β-loop lead to progressive destabilization of the protein. Furthermore, the thermal unfolding transitions characteristic for thumb site II inhibitor – NS5B complex are absent in the inhibitor – bound constructs in which interactions between C-terminal tail and β-loop are abolished, pointing to the pivotal role of both regulatory elements in communication between domains. Taken together, a comprehensive picture of inhibition by compounds binding to thumb site II emerges: inhibitor binding provides stabilization of the entire polymerase in an inactive, closed conformation, propagated via coupled interactions between the C-terminal tail and β-loop.

## Introduction

Hepatitis C virus (HCV), a member of the *Flaviridae* family, is a positive single-strand RNA virus. An estimated 3% of world's population is chronically infected with HCV, with 30% of carriers expected to develop serious liver-related diseases, including hepatocellular carcinoma, over a period of 10 to 30 years [1]. Over the last decade there has been an ongoing effort to develop new direct acting antivirals (DAA) to improve the therapeutic outcome of anti-HCV treatment [2], [3]. Anti-HCV DAAs currently in development target the non-structural viral proteins, with many focused on inhibition of NS5B [4], [5]. HCV NS5B functions as an RNA dependent RNA polymerase (RdRp) and is the catalytic component of the HCV replication complex built of multiple HCV non-structural proteins and host factors. NS5B transcribes viral RNA for protein translation and progeny genome production [6]. Since mammalian cells lack an RdRp polymerase equivalent, HCV NS5B is an attractive target for development of small molecule inhibitors with the potential to selectively inhibit viral replication.

**Figure 1:**
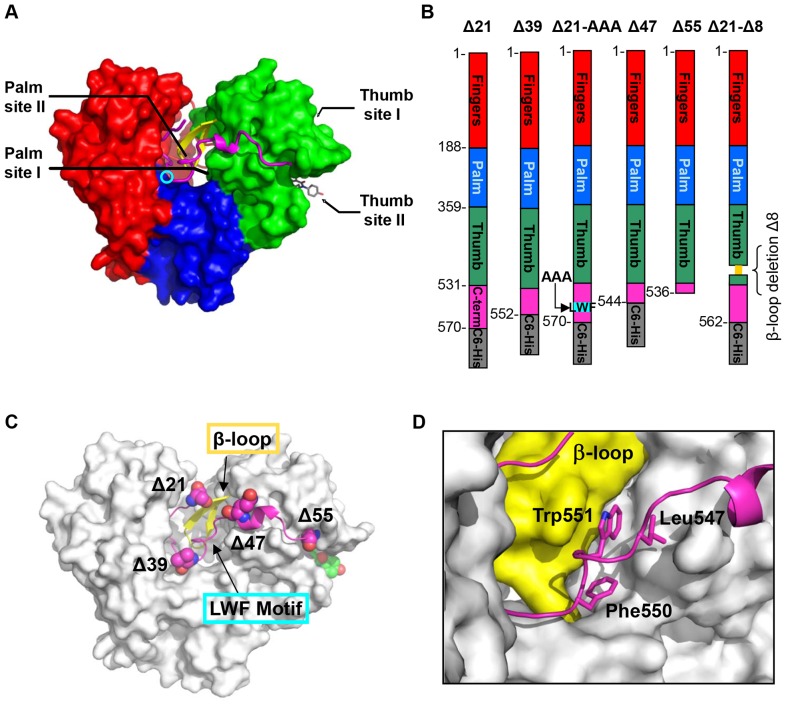
HCV NS5B polymerase nonnucleoside inhibitors binding sites and NS5B constructs used in studies. (**A**) Thumb site I and thumb site II are located on the thumb domain (green); palm site I and palm site II are at the interface of the three domains, thumb, palm (blue) and fingers (red). GS-9669 inhibitor bound in the thumb site II pocket is shown in stick representation (grey, description of crystal structure of NS5B bound to thumb site II inhibitor GS-9669 will be published elsewhere). The active site is indicated by the cyan circle. The other main structural features shown are the C-terminal tail residues (magenta) which contact the β-loop (yellow). (**B**) 2D representation of domain structure of polymerase and C-terminal truncation sites Δ21, Δ39, Δ47, Δ55, as well as the β-loop deletion mutant Δ21-Δ8 (deleted residues shown in yellow) and LWF triple A mutant F550A/W551A/L553A. Δ55 is a tag free construct and all others contain C6-His. (**C**) Location of the mutations relative to the tertiary protein structure. (**D**) Close-up view of interface between LWF motif (magenta, stick representation) and β-loop (yellow) which is dominated by hydrophobic contacts on the surface of the protein.

There are two major classes of NS5B inhibitors: active site nucleotide/nucleoside analogues (Nucs), and nonnucleoside inhibitors (NNIs) that bind to allosteric sites on the enzyme. Nuc inhibitors mimic the natural substrates and function as chain terminators by incorporation into viral RNA. In contrast to Nucs, the NNIs are thought to achieve NS5B inhibition by affecting conformational states of the protein. Crystal structures of NS5B in complex with NNIs, together with resistance analysis, have revealed at least four pockets as allosteric inhibitory sites (Figure 1A).

Despite a wealth of enzymatic and structural information regarding HCV NS5B [7], [8], [9] there are gaps in our understanding of the precise mechanism of RNA replication and the conformation and dynamics of the protein necessary to support RdRp activity. The recombinant proteins that have been studied are truncated forms of the full length protein with the C-terminal transmembrane helical segment and some additional C-terminal residues removed. The most common is the NS5B Δ21 mutant, with the C-terminus ending at residue 570 and a 6-His tag added (NS5B_570_, Figure 1B). The structure of NS5B, first determined in 1999, is similar to most polynucleotide polymerases [8], [10] and consist of three subdomains (Figure 1A): the palm domain (blue), which serves as the foundation for the active site, the finger domain (red) and the thumb domain (green). HCV NS5B also displays structural characteristics unique to this viral RdRp class. The finger domain contains extended loops, named Λ1 and Λ2, which reach over and interact with the top of the thumb domain to envelop the active site [9], [11], [12]. In addition, a structural motif termed the β-loop (residues 443–455) extends from the thumb domain into the active site (Figure 1C). The residues of the C-terminal tail remain in intimate contact with the thumb (β-loop), finger and palm domains [7] (Figure 1C and D).

It has been postulated that the vast majority of crystallographically observed conformations of NS5B, including all inhibitor bound structures, represent a closed, inactive form of the enzyme [11], [12], [13], [14]. In this closed conformation, the polymerase Λ1 finger loop maintains contact with the thumb domain, the β-loop is positioned between finger and thumb domains preventing double stranded RNA egress, and the C-terminal tail is located on top of the β-loop. This closed conformation does not preclude binding of short single stranded template RNA [11], [15], [16], [17]. However, the channel is too narrow (6 to 20 Å) to accommodate duplex RNA [7], [8]. Several studies have demonstrated the importance of thumb domain contacts with the C-terminal tail [10], the Λ1 loop, and/or the β-loop, in the regulation of RdRp activity [14], [16], [18]. It was proposed therefore that enzyme inhibition by all allosteric DAAs is most likely accomplished by preventing conformational transitions required for initiation and/or product elongation [19], [20], [21], [22].

Several potential conformations of NS5B that would allow binding of double stranded RNA in the active site and polymerization have been proposed, including a large rotation of the thumb domain [7], [8], [12], [21], [23], but the structure of the open conformation remains an enigma. The recently published structure of duplex RNA in complex with NS5B possessing β-loop deletion [24] gives an interesting snapshot of a potential path from *de novo* initiation to productive elongation. While this structure has a more open active site as a result of β-loop truncation and movement of the thumb and fingers domains away from each other to accommodate the double stranded RNA, the primer and template do not seem to be located in a position that would allow proper elongation, when compared to structures of other polymerases (HIV reverse transcriptase, poliovirus, Norwalk virus). A model that incorporates the unique structural characteristics of the HCV polymerase is required to explain the activity of inhibitors that may prevent the transition to the open form.

In this study we characterize the mechanism of action of GS-9669, an allosteric inhibitor of NS5B that binds to the thumb site II and is at present in phase II clinical trials in HCV patients. Our approach relies on enzyme inhibition, calorimetric, and direct binding studies with the use of GS-9669 and two other thumb site II inhibitors, along with other NNIs. We also utilize several truncation mutants of HCV NS5B that disrupt critical interactions at the interface of the thumb domain, β-loop and C-terminus (Figure 1B–D) to investigate possible conformational communication pathways within NS5B polymerase. Our studies demonstrate unique binding and inhibition profiles for each NNI class across mutant constructs. An intact interface between the β-loop and the C-terminus is required for inhibitory potency of GS-9669, although both elements are dispensable for inhibitor binding. Binding of thumb site II inhibitors leads to stabilization of NS5B in the closed conformation, facilitated by the interaction between these regulatory elements.

## Methods

### Inhibitors

**Figure 2:**
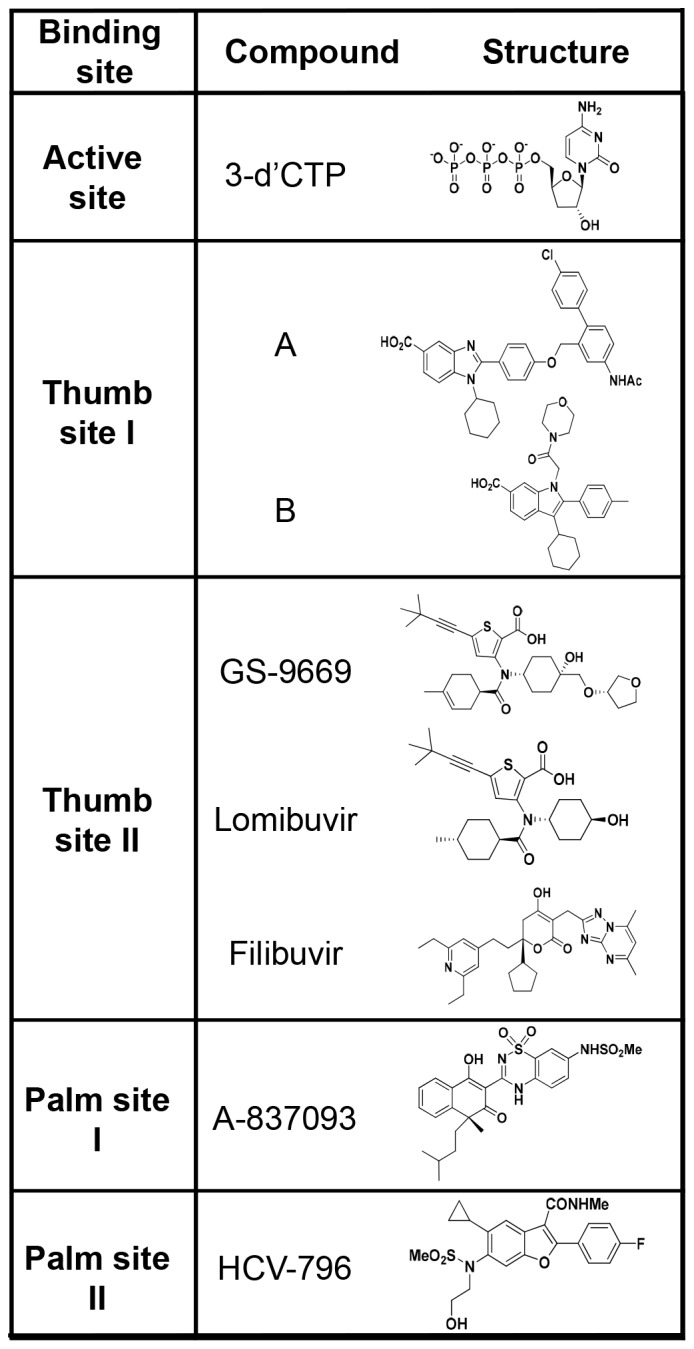
Structures of HCV NS5B active site nucleotide inhibitor and NNIs targeting four allosteric sites used in this study.

Unless specified otherwise representative inhibitors for the four allosteric sites and active site nucleotide inhibitor used in this study (Figure 2) were synthesized at Gilead Sciences Inc. (Foster City, CA). Thumb site I-A [25], and thumb site I-B [26] were synthesized at BioArc Research Solutions, Gujarat, India. Three different thumb site II inhibitors were used: GS-9669 [27], lomibuvir [28] (synthesized at Curragh Chemistries, Cleveland, OH) and filibuvir [29]. Palm site I, A-837093 [30] was synthesized at Shanghai Medicilon Inc. (Shanghai, China). Palm site II, HCV-796 [20] was synthesized at Curragh Chemistries. Active site inhibitor 3′dCTP was purchased from Trilink BioTechnologies (San Diego, CA).

### Cloning of the Δ21 (NS5B_570_), Δ39 (NS5B_552_), Δ47 (NS5B_544_), Δ55 (NS5B_536_), Δ21-AAA (NS5B_570-LWF_) and Δ21-Δ8 (NS5B_562_) GT1b Con-1 NS5B constructs

The coding sequences of GT1b (con-1 strain) NS5B polymerase with various truncations at the carboxyl terminus were PCR amplified from a plasmid encoding the I389luc-ubi-neo/NS3-3′/ET replicon [31]. The 3′-PCR primers were designed to encode a hexahistidine tag and to amplify a sequence terminating at the amino acid residues at position 570, 552, 544 and 536 of the GT1b-con1 NS5B gene, respectively. The resulting PCR fragments were inserted into pET21a protein expression vector (EMD BioSciences, Darmstadt, Germany), yielding plasmids that expressed NS5B(1b) Δ21-C6His, NS5B(1b) Δ39-C6His, NS5B(1b) Δ47-C6His and NS5B(1b) Δ55-C6His. The coding sequence that corresponds to 1–536 amino acid residues of GT1b con-1 NS5B was PCR amplified and cloned into pET-SUMO plasmid by TA-cloning® according to manufacturer's protocol (Life Technologies, Carlsbad, CA) to generate the construct that expresses SUMO-NS5B Δ55 (GT1b). To generate the triple alanine mutations at NS5B positions L547, W550 and F551, a pair of oligonucleotide primers which incorporated the codon change based on the template pET 21-NS5B(1b)Δ21-C6His was designed and mutagenesis was performed using QuikChange®II XL Site-directed Mutagenesis kit. The sequence of NS5B Δ21-Δ8 (GT1b) was based on the crystallography construct PDB entry 1C2P [9] which was expressed as an N-terminal hexahistidine tag protein. A pair of mutagenic phosphorylated oligonucleotides was designed that annealed to NS5B residues 443 and 455 in the reverse and forward direction, respectively, and the PCR reaction was performed using the Phusion Site-directed mutagenesis kit (New England BioLabs, Ipswich, MA) to remove and replace amino acid residues 444 to 454 with two glycine residues.

### Expression of GT1b Con-1 NS5B constructs

BL21(DE3) bacteria were transformed with the NS5B polymerase expression vector pET21a-NS5B(1b) Δ21 C6His, and a 200 mL overnight LB culture was inoculated into a 20 L fermentation vessel (Sartorius BBI System Inc., Bethlehem, PA), containing 18 L of fresh 2xYT medium supplemented with 100 µg/mL ampicillin. When cell densities reached an OD_600_ of 1, the culture temperature was reduced from 37°C to 25°C and induction was immediately initiated by the addition of IPTG to a final concentration of 0.5 mM. After a three hour induction, cells were harvested by centrifugation and stored as frozen pellets at −80°C.

### Purification of the Δ21 (NS5B_570_), Δ39 (NS5B_552_), Δ47 (NS5B_544_), Δ55 (NS5B_536_), Δ21-AAA (NS5B_570-LWF_) and Δ21-Δ8 (NS5B_562_) GT1b Con-1 NS5B constructs

Cell pellets were thawed and resuspended at 10 mL/g cells in Buffer A (50 mM Tris-HCl, pH 7.6, 300 mM NaCl, 10% glycerol, 3 mM β-mercaptoethanol and 2 mM PMSF). The cell suspension was homogenized, filtered through cheesecloth, and passed three times through a microfluidizer (Microfluidics, model 110Y, Newton, MA) at 18,000 psi. The resulting lysate was centrifuged at 15,500 rpm for 45minutes and the lysate supernatant was loaded onto a Ni-HiTrap column (GE Healthcare, Piscataway, NJ, cat # 170-5248-02) pre-equilibrated with five column volumes of the Buffer A. Proteins were eluted with a buffer containing 50 mM Tris-HCl, pH 7.6, 300 mM NaCl, 10% glycerol, 3 mM β-mercaptoethanol, and a 25–500 mM imidazole-HCl gradient. Fractions containing NS5B protein were collected, pooled, and dialyzed overnight at 4°C into 50 mM Tris-HCl, pH 7.0, 50 mM NaCl, 10% glycerol, 2 mM dithiothreitol. The sample was then diluted 1 4 with Buffer B (50 mM Tris-HCl, pH 7.0, 10% glycerol, 2 mM dithiothreitol) and loaded onto a SP cation exchange column (GE Healthcare, cat #17-5157-01) equilibrated in Buffer B. Recombinant NS5B protein was eluted with 0–1 M NaCl gradient in Buffer B. The enzyme was stored at −80°C in a buffer containing 50 mM Tris-HCl, pH 7.0, 600 mM NaCl, 10% glycerol, 2 mM dithiothreitol.

### Purification of SUMO GT1b NS5B ΔC55

Once the cell pellet was lysed the resulting cell lysate was then loaded onto a Ni HiTrap column (GE Healthcare, cat # 170-5248-02) pre-equilibrated with Buffer A and eluted as described previously. To the pooled fractions (NS5B concentration of ∼0.5 mg/mL in 15 mL) 2 mL Sumo Protease was added to obtain a 1 10 (w/w) ratio of protease to NS5B and dialyzed overnight at 4°C against 4 L of 25 mM Tris-HCl, pH 7.8, 250 mM NaCl, 10% glycerol, 3 mM β-mercaptoethanol. The dialysate was centrifuged at 15,000 rpm for 20minutes and the soluble portion was decanted, loaded onto a Ni HiTrap column pre-equilibrated with 2 column volumes of Buffer A, the flow-through fraction was collected and concentrated to 1 mL prior to loading onto a size exclusion column (KW2003, Shodex, New York, NY) pre-equilibrated with 100 mL buffer 50 mM Tris-HCl, pH 7.0, 600 mM NaCl, 10% glycerol, 2 mM DTT. Eluted fractions containing NS5B Δ55 were pooled and concentrated to 4 mg/mL, flash-frozen and stored at −80°C.

### HCV NS5B Model with duplex RNA

Poliovirus polymerase (3OL7) [32] and HCV NS5B 2a Δ21-Δ8 (4E7A) [24] structures were overlaid based on Cα superposition of active site residues. Primer/template, metals, and substrate from the poliovirus structure were merged into the NS5B structure. Minimization of some amino acids side chains surrounding the RNA was required. Metals were modified from Mn^2+^ to Mg^2+^. Active site side chains (particularly D225 and S282) were manipulated into conformations consistent with the Norwalk structure (3BSO) [33] and poliovirus and minimized, constraining the coordination of the metals (MacroModel, OPLS-AA/GB/SA, Schrödinger, San Diego, CA). Additional amino acid side chains were conformationally sampled using Prime Schrödinger software suite 2011. The β-loop was manually incorporated and optimized with dynamics (MacroModel). Finally, the structure was mutated to genotype 1b Con1 and side chains were again optimized with Prime.

### HCV NS5B polymerase inhibition assay

The HCV NS5B polymerase assay measures the incorporation of ^33^P-CTP into an RNA product using a heterologous RNA template. The assay was performed at multiple inhibitor concentrations to determine inhibitor concentration resulting in 50% inhibition of enzymatic activity (IC_50_). The compound was serially diluted 3-fold in 100% DMSO from a starting DMSO stock concentration of 4 mM, and then diluted 1 40 in the assay buffer containing 50 mM Tris-HCl, pH 7.5, 10 mM KCl, 5 mM MgCl_2_, 1 mM EDTA, 1 mM dithiothreitol. Finally, 4 µL of this dilution was added to a 96 well reaction plate containing 25 nM NS5B Con1 constructs and 4 ng/µL RNA template (heteropolymer designed to be secondary structure free [34]) for a total volume of 32 µL. Compounds were pre-incubated with NS5B for 15minutes at room temperature. The reaction was initiated with addition of 250 µM ATP, GTP, UTP, 200 nM CTP, 5 nM/0.6 µCi ^33^P-CTP (PerkinElmer, Waltham, MA) and incubated for 60minutes at 30°C. The total reaction volume was 40 µL with 0.25% DMSO final concentration. The reaction samples were transferred to a 96 well white Whatman Unifilter 350 plate (Millipore catalog# 7700-4313, Billerica, MA), washed three times with 125 mM sodium phosphate dibasic, once with water, once with 100% ethanol and then air-dried. 100 µL of Microscint-20 (PerkinElmer) scintillation fluid was added to each well and the plate was read on the Top Count scintillation reader (PerkinElmer). Data were further processed using the following protocol: raw data points were normalized by subtraction of the average background control and divided by the average DMSO control, yielding % Activity compared to DMSO. Duplicate measurements were averaged and the mean values and standard deviations were used in the fitting procedure. The IC_50_ was determined by applying the Sigmoidal Dose Response Model equation, fit  =  A+((B-A)/(1+(X/C)?D)), where A is the lowest % Activity, B is the highest % Activity, C is the IC_50_, D is a Hill slope and X is an inhibitor concentration. The 10 point dose response curves were generated in XLFit (IDBS, Guildford, UK), using model 205.

### SPR affinity assay

All SPR experiments were performed on a Biorad ProteOn XPR36 using GLH chips (BioRad, Hercules, CA). With distilled water as running buffer, each chip was pre-conditioned using a 1-minute pulse each of 0.5% SDS, 50 mM NaOH, 100 mM HCl, and 10% DMSO in both the horizontal and vertical directions. Channels were activated with a 5-minute injection of EDC/sulfo-NHS in running buffer composed of 50 mM HEPES, pH 7.5, 5 mM MgCl_2_, 10 mM KCl, 1 mM EDTA, 1 mM TCEP, and 0.01% P-20. This was followed by immobilization of the NS5B constructs Δ21, Δ55, and Δ21-Δ8 in designated channels of the GLH chip. Prior to immobilization, constructs were diluted in 10 mM HEPES, pH 7.5, 150 mM NaCl. This was considered to be a more gentle buffer than the low pH acetate buffers containing no salt traditionally used to chemically immobilize proteins. Gentler immobilization conditions were chosen in an attempt to maximize the activity of the Δ55 and Δ21-Δ8 surfaces. Final immobilization levels ranged between 9000–11000 RU. Surfaces were deactivated by a 5-minute injection of ethanolamine. The L1 channel served as a negative control surface and was activated and deactivated with no protein immobilized. Compounds were prepared by first serially diluting in 100% DMSO. Samples were then transferred into running buffer containing no DMSO to yield the desired concentration of compound and a final DMSO concentration of 5% (matching the final DMSO concentration in the running buffer). A DMSO concentration series (ranging from 4.5% to 5.5% DMSO) was also injected so that a DMSO calibration curve to correct for excluded volume effects could be generated. Compounds were run at a flow rate of 100 µL/min and for a contact time of 90seconds. Dissociation times varied for different compounds and ranged between 1 and 90minutes. Data were analyzed using ProteOn software (BioRad). Sensorgrams were double-referenced and corrected for excluded volume effects and then fitted to a simple kinetic 1 1 binding model yielding the on- and off-rate constants *k_a_* and *k_d_*, the equilibrium dissociation constant K_D_, as well as the theoretical maximal response (R_max_).

### NS5B polymerase Differential Scanning Fluorimetry assay

Differential Scanning Fluorimetry (DSF) measures the midpoint of unfolding transition of protein – T_m_ – during thermal denaturation based on the change in fluorescence intensity of environmentally sensitive dye Sypro Orange (Life Technologies, Grand Island, NY) [35], [36]. DSF was carried out in 25 µL reaction volume on a ViiA7 Real-Time PCR (Life Technologies) in buffer containing 50 mM Hepes, pH 7.0, 200 mM KCl, 2.5% glycerol, 1 mM TCEP, 1 mM MgCl_2_, 1 mM EDTA, 1.5% DMSO. The assay was performed in the absence and the presence of compounds. Various constructs of NS5B at ∼5 µM concentration were preincubated with 1.5% DMSO or inhibitor at 25 µM for 5minutes at room temperature. To prevent bleaching 1 1000 diluted Sypro Orange fluorophore was added to the reaction mixture just prior to thermal denaturation. All concentrations were final after mixing. Thermal denaturation was carried out within 5–10minutes of dye addition by increasing the temperature from 25°C to 95°C with 0.017°C/second ramp. Fluorescence intensity data (excitation at 490 nm and emission at 575 nm) were collected at 0.07°C intervals and analyzed with Protein Thermal Shift Software (Life Technologies) using the first derivative approach to calculate T_m_. For each compound delta T_m_ (ΔT_m_) was calculated by subtracting the T_m_ value obtained for protein alone (apo) from the T_m_ value obtained in the presence of compound (bound).

### NS5B polymerase Differential Scanning Calorimetry (DSC) assay

All DSC measurements were performed on a Microcal VP-Capillary DSC platform (GE Healthcare). Protein samples were dialyzed overnight at 4°C in a buffer composed of 50 mM Hepes, pH 7.0, 200 mM KCl, 1 mM MgCl_2_, 1 mM EDTA, 1 mM TCEP, 5% glycerol. The protein was then diluted in dialysate to a final concentration of 5 µM and compound (10 mM DMSO stock or DMSO alone as a control) was added to a final concentration of 25 µM yielding 1.5% final concentration of DMSO. Matched dialysate buffer was used as a reference. The samples were scanned from 10°C–85°C at a rate of 60°C/hour using a filtering period of 8seconds and with 15minutes of pre-equilibration prior to the start of each run. Data were first normalized for protein concentration, then baseline corrected and buffer subtracted using Origin 7.0 AutoSampler DSC software (GE Healthcare). Melting transitions were irreversible and were analyzed with cursor initiated DSC peak fit function using the non-two- state unfolding model within the Origin 7.0 software to enable deconvolution of multiple transitions, determination of the relative change in enthalpy (ΔH) for the whole melting transition of each sample and for individual transitions, and the apparent thermal unfolding midpoint (T_m_ apparent) for each transition. ΔH was estimated from the area under the curve by integrating the change in the heat capacity with respect to the temperature (Origin 7.0, model dependent enthalpy). %ΔH for the deconvoluted transitions was calculated as the % of Area for each peak with respect to the total ΔH, or total Area, for the entire melting transition for each sample [37].

## Results and Discussion

### Structural insights from model of the open, active HCV NS5B polymerase

**Figure 3:**
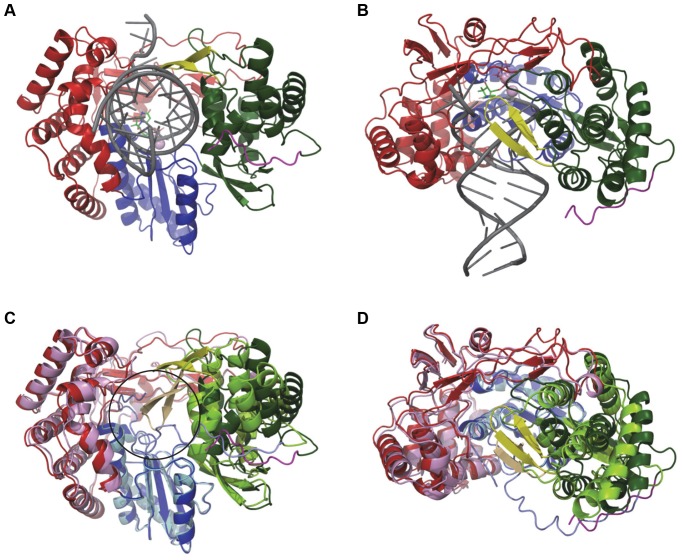
Model of HCV NS5B bound to duplex RNA in elongation mode. Front (**A**) and side view (90° rotation with cutaway through the finger domain) (**B**) of NS5B bound to primer/template RNA (dark grey). Side view shows how the β-loop wraps around RNA as it exits the polymerase. Front (**C**) and side (**D**) view of the overlay of NS5B Δ21 structure (fingers-pink, palm-light blue, thumb-light green, PDB entry 1C2P) with the model. Overlay shows that the transition from closed inactive polymerase to elongating enzyme requires only small expansion of the finger (red), palm (blue) and thumb (green) domains and major displacement of the β-loop (yellow) and C-terminal tail (magenta). Black circle in (C) denotes the position of double stranded RNA in the model.

To obtain additional insights into the conformational changes involved in the transition of NS5B from closed to open form, we built a model of elongating genotype 1b NS5B based on the primer-template bound structure published by Mosley *et al*. [24] and modified to be catalytically competent based on the structure of poliovirus and Norwalk virus polymerases. The structural changes required to bind the RNA product (Figure 3A–D) are in contrast with earlier reports that proposed both a large rotation of the thumb domain and a detachment of the Λ1 loop [8], [11], [38], [39]. In our model, the Λ1 loop maintains contact with the thumb domain. Overall, the palm domain remains essentially unchanged (RMSD  = 0.5 Å) while the fingers move ∼1 Å and the thumb moves by ∼2 Å. In particular, the largest structural rearrangements occur at the interface of the C-terminal tail/thumb domain and the β-loop/C-terminal tail, which form the moving parts that rearrange to accommodate RNA product.

This model is in good agreement with mutational data for thumb site II inhibitors, which mapped viral resistance mutations towards this class of compounds to the vicinity of the β-loop as well as to the binding [13], indicating the importance of the former as a key allosteric regulatory component of polymerase activity and implicating the involvement of disruption of its interactions in the transition to the open conformation. The NS5B Δ21 polymerase is also tolerant of further C-terminal truncations [10], with several of the most truncated constructs actually possessing higher enzymatic activity relative to Δ21. The same is true for all constructs used in this study. Δ21-AAA and Δ47 displayed 350% and 390% higher RdRp activity, respectively, and Δ55 and Δ21-Δ8 were 1000% more active than Δ21 as measured by a radiometric filter binding assay (data not shown and File S1). The genotype 2a Δ21-Δ8 mutant construct, which has close sequence and structural homology to NS5B 1b genotype, also shows increased activity [24]. The increased activity upon removal of the β-loop and C-terminal tail residues is consistent with their role as key RdRp regulatory elements, resulting in an artificially “open” polymerase conformation that favors productive elongation similar to that observed in the related Norwalk virus and poliovirus polymerase - RNA complex structures [32], [33]. This observation is in agreement with molecular dynamics simulations performed on both the Δ21 and Δ55 NS5B constructs, which showed that in the presence of the terminal 21 residues the polymerase dynamics and sampling of the initiation competent conformations was severely restricted [40].

### Potency of allosteric site inhibitors against NS5B polymerase Δ21, β-loop deletion and C-terminal truncation mutant constructs

**Figure 4:**
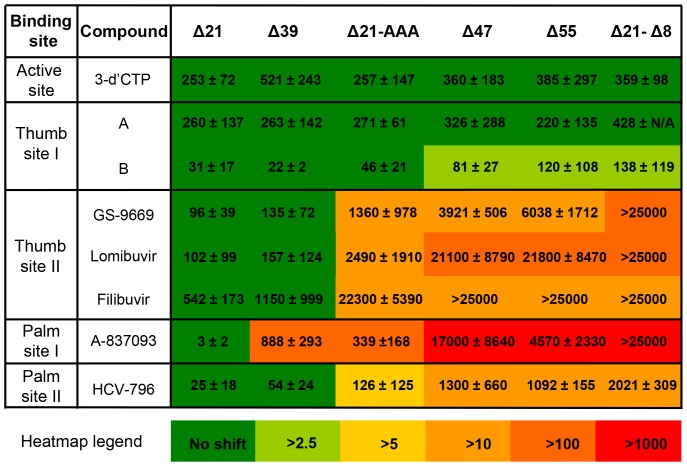
IC_50_ values for inhibition of RdRp activity of NS5B by Nuc (3′-d′CTP) and NNIs. Numbers represent mean and standard deviation of IC_50_ values determined for each inhibitor against the set of NS5B constructs. Heatmap is colored according to IC_50_ fold shift relative to Δ21 to show changes in inhibition profile for given NNI across NS5B mutant constructs. Thumb site II inhibitors begin to show significant loss of inhibitory potency as interactions between β-loop and C-terminal residues are disrupted by mutations in the interface (Δ21-AAA, truncations past Δ39 and Δ21-Δ39 mutations). Palm site I inhibitor is affected as well, which is explained by disruption of the inhibitor's interaction with the β-loop and C-terminal residues. Inhibition by thumb site I remains for the most part unaffected by NS5B mutations whereas inhibition by palm site II NNI is reduced due to decrease in binding to NS5B with truncated β-loop and/or C-terminal.

Inhibitor potencies were compared in an enzymatic, template-directed RNA primer elongation assay for a representative set of the four classes of allosteric inhibitors and the following NS5B polymerase constructs: the canonical Δ21 (NS5B_570_), Δ39 (NS5B_552_), Δ47 (NS5B_544_) and Δ55 (NS5B_536_) C-terminal truncations; Δ21-AAA (NS5B_570-LWF_), in which hydrophobic residues of the C-terminal LWF motif that interact with the β-loop in the closed conformation were removed; and the β-loop deletion Δ21-Δ8 (NS5B_562_) (Figure 1, Figure 4 and Figure S1).

The three thumb site II inhibitors effectively inhibited RNA elongation by Δ21 (Figure 4), and retained similar potency towards Δ39, which still contains the hydrophobic LWF motif. Activity against the other C-terminal truncation variants and construct with LWF motif mutated was reduced, and the β-loop deletion construct was completely insensitive to inhibition - providing evidence for the importance of interactions between the β-loop and the C-terminal tail in the mechanism of inhibition through small molecules binding to thumb site II.

In contrast, the potency of control inhibitors 3′dCTP (a nucleotide NS5B chain terminator) and two thumb site I NNIs remained unchanged against all the constructs (Figure 4). The palm site I inhibitor exhibited reduced potency towards Δ39 and Δ21-AAA, and was only marginally active against Δ47, Δ55 and Δ21-Δ8. The palm site II inhibitor displayed low double digit nanomolar inhibition of Δ21 and retained submicromolar to low micromolar activity against all constructs, but it had reduced potency in those containing modifications to the β-loop and/or its interactions with the C-terminal tail.

### Direct binding of Site I–IV allosteric inhibitors to the panel of NS5B mutant constructs

To determine if loss of inhibition was simply due to decreased affinity of the inhibitors to the mutant NS5B constructs, Surface Plasmon Resonance (SPR) measurements were conducted for Δ21, Δ55, and Δ21-Δ8 (Table 1, Table S1 in File S1 and Figure S2). No significant change in the equilibrium dissociation constant (K_D_) for binding of the thumb site I inhibitor to Δ55 and Δ21-Δ8 construct relative to Δ21 was observed in agreement with potent inhibition of enzymatic activity of NS5B mutant proteins by this class of compounds. For the palm site I inhibitor, a large increase in K_D_ for binding to Δ55 relative to Δ21 was observed, in agreement with the profoundly weaker inhibitory potency against this construct. A K_D_ for palm site 1 inhibitor binding to Δ21-Δ8 could not be determined due to super-stoichiometric responses observed at the higher concentrations of compound that were required to detect signal on this surface. This indicates that the high affinity binding observed for this compound on the Δ21 surface has at best been reduced to very weak association. This data is also consistent with reduced inhibitory potency of the palm site I inhibitor against Δ21 and diminished activity for Δ21-Δ8.

**Table 1:** Equilibrium dissociation constants (K_D_) for binding of NNIs to Δ21 and fold-shifts in K_D_ for association to Δ55 and Δ21-Δ8 determined by SPR.

		K_D_ (nM)^a^	Fold shift from Δ21^b^	
Site	Inhibitor	Δ21	Δ55	Δ21-Δ8
Thumb site I	B	11.7	1.0	1.6
Thumb site II	GS-9669	1.6	5.5	1.7
Thumb site II	Lomibuvir	3.3	9.0	2.0
Thumb site II	Filibuvir	38.2	9.3	2.4
Palm site I	A-837093	0.04	433	n/a^c^
Palm site II	HCV-796	174.0	n/a^c^	n/a^c^

^a^ Sensorgrams for the binding of NNIs to Δ21, Δ55 and Δ21-Δ8 along with a table of equilibrium and kinetic parameters of interaction are shown in Figure S2 and Table S1 in File S1, respectively.

^b^ Fold shift in K_D_ for association of NNIs to Δ55 and Δ21-Δ8 was calculated relative to the K_D_ determined for binding towards Δ21.

^c^ Where K_D_ values could not be obtained either due to complex binding kinetics (Palm Site II inhibitor binding to Δ55 and Δ21-Δ8) or super-stoichiometric binding (Palm Site I inhibitor binding to Δ21-Δ8), numerical values have been replaced by n/a (not applicable).

Binding of the palm site II inhibitor to Δ55 was marked by significant deviations from simple 1 1 kinetics. The origin of this complexity is puzzling given the unequivocal fitting of sensorgrams collected for the thumb site II inhibitor filibuvir to a simple 1 1 model on all three surfaces. Rather than fit the binding data of the palm site II inhibitor to Δ55 and Δ21-Δ8 using a complex kinetic model, we note a qualitative differences in the sensorgrams obtained for those two protein constructs: namely, an obvious disappearance of a single slow dissociation component that is clearly present in sensorgrams collected for the Δ21 surface, suggesting a decrease in affinity of palm site II inhibitor towards Δ55 and Δ21-Δ8. Such a reduction in affinity is consistent with the 44- to 81-fold weaker inhibitory potency of the palm site II inhibitor against Δ55 and Δ21-Δ8, respectively, in comparison to Δ21. In contrast, thumb site II compounds show only a modest reduction in affinity for the Δ55 and Δ21-Δ8 constructs despite dramatic reductions in inhibitory potency. Thus, among the NNIs the thumb site II inhibitors display a unique profile: disruption of the interaction between the LWF motif and β-loop in the thumb domain by deletion or mutation has little to no impact on inhibitor binding, yet leads to significant loss of inhibitory potency. This indicates a critical role of both structural elements in communication between the inhibitor - occupied thumb site II and the NS5B catalytic site.

### Measurement of NS5B mutant constructs stability and binding of thumb site II inhibitors by DSF

**Figure 5:**
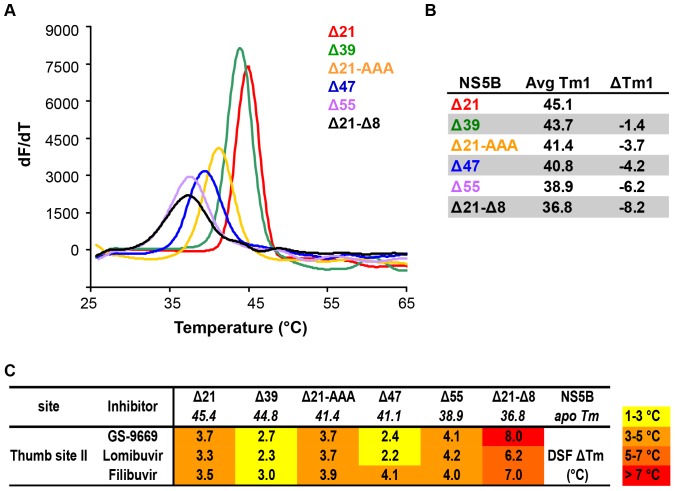
Effect of mutations in structural motifs (C-terminus, β-loop) of NS5B on protein stability and inhibitor binding. (**A**) Representative DSF melting profile for apo NS5B mutant constructs at 5 µM protein concentration are shown. Trend in stability is: Δ21 > Δ39 >> Δ21–AAA ≈ Δ47 > Δ55 > Δ21-Δ8. (**B**) DSF average T_m_ and T_m_ shifts relative to apo Δ21 (ΔT_m_) for mutant constructs. (**C**) Direct binding of NNIs to NS5B constructs determined by upshift of NS5B T_m_ in the presence of 25 µM concentration of thumb site II inhibitor. Values represent an average of two or more independent experiments (each run in triplicate) with standard deviations within 0.5 to 1.0°C, unless mentioned otherwise. Heatmap is colored according to the magnitude of ΔT_m_ elicited by inhibitor binding. Thumb site II inhibitors show >2°C T_m_ upshift for all constructs in agreement with SPR data obtained for selected constructs.

To explore how the various modifications of NS5B influence its thermal and conformational stability and to understand the relationship between thumb site II inhibitor binding and protein construct stability, differential scanning fluorimetry was employed. Figure 5A–B shows the thermal unfolding transitions and an average absolute T_m_ for each construct along with the change in T_m_ relative to apo Δ21 (ΔT_m_). With successive removal of key structural elements involved in the regulation of RdRp activity, a progressive decrease in the midpoint of thermal unfolding temperature relative to Δ21 is observed (Figure 5B), consistent with the gradual decrease in contacts made between the C-terminal tail residues and the β-loop, which are thought to be stabilizing interactions [10], [12], [38].

The impact of binding of thumb site II inhibitors on the mutant constructs was explored by determining the changes in T_m_ of each construct in the presence of inhibitor (25 µM compound concentration; 5-fold molar excess over protein) relative to apo protein. Stabilization of a folded form of a protein by small molecule binding, and the attendant T_m_ upshift (ΔT_m_), derives from the contribution of the free energy of binding to the free energy of (un)folding according to a two-state unfolding model [41]. The data are presented as a heatmap in Figure 5C.

In the presence of the thumb site II inhibitors GS-9669, lomibuvir, and filibuvir there is an increase in T_m_ for all NS5B mutant constructs relative to the Tm for apo protein, showing that this class of inhibitors retains the ability to bind and stabilize all protein constructs. This is consistent with the SPR binding data for Δ21, Δ55, and Δ21-Δ8. Interestingly, the T_m_ upshift is greatest for the destabilized Δ55 and Δ21-Δ8 constructs, despite the lack of inhibition of their enzymatic activity by this inhibitor class. This indicates that binding and stabilization of the polymerase in the absence of the C-terminal tail or the β-hairpin is not sufficient to confer inhibition of RdRp activity by the thumb site II inhibitors.

### Binding and conformational profiling by Capillary Differential Scanning Calorimetry

Differential scanning calorimetry (DSC) was employed for a subset of constructs and construct – inhibitor pairs to understand the relationship between the inhibitor binding and protein thermal and conformational stability. Excellent agreement between melting temperatures determined by DSC and DSF for apo Δ21, Δ39 and Δ47 proteins was observed (Figure S3A and B). In addition to determination of the absolute T_m_ and relative T_m_ shifts that are measurable by DSF, DSC also allows for the deconvolution of multiple transitions of the protein as it goes from the folded to the unfolded state. Although NS5B undergoes an irreversible non-two-state melting transition, all DSC experiments were performed under identical conditions allowing for comparison of thermal unfolding profiles, relative stabilities and apparent T_m_.

**Figure 6:**
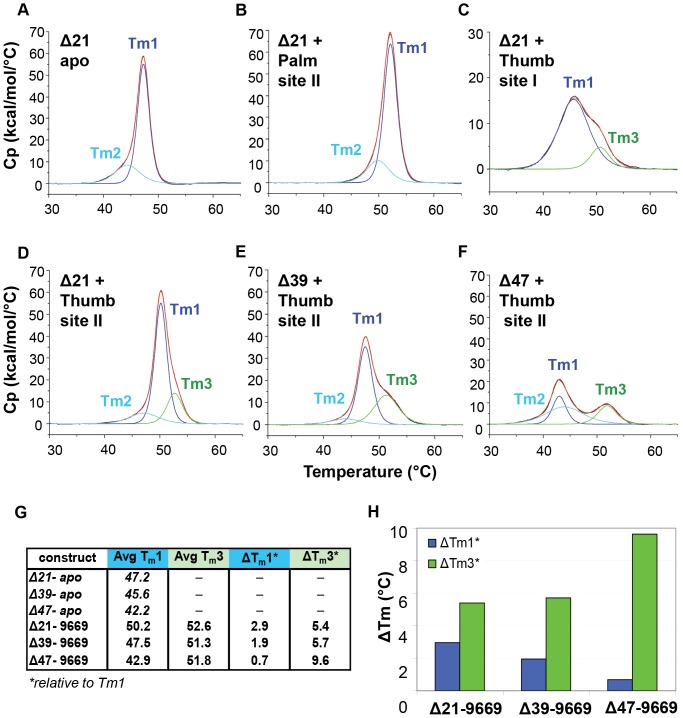
Binding of thumb domain NNIs to NS5B results in appearance of a new transition with T_m_3. (**A**) Apo Δ21 thermal unfolding profile (black line) shown with fit to a non-two-state unfolding model (red) with two transitions: major with T_m_1 (dark blue line) and leading shoulder with T_m_2 (light blue line); (**B**) Thermal unfolding of Δ21 bound to palm site I shows the same profile as observed for apo Δ21. Melting profile of apo Δ21 bound to palm site II inhibitor is similar to the one observed for Δ21-palm site I complex and apo protein (not shown); (**C**) Thermal unfolding of Δ21 bound to thumb site I inhibitor with fit to a non-two-state model (red line) with two transitions: major with T_m_1 (dark blue line) and a new transition with T_m_3 (green line); (**D**) Unfolding profile of Δ21 bound to thumb site II inhibitor GS-9669 shows three transitions. T_m_3 is analogous to the midpoint of second transition observed with thumb site I. (**E**) and (**F**) Melting profiles for Δ39 and Δ47 bound to thumb site II inhibitor. (**G**) Average T_m_3 for the third transition in constructs with C-terminal tail truncations. Value of T_m_3 remains constant with standard deviation better than 0.5°C, but ΔT_m_3 increases with increasing C-terminal truncations. (**H**) Comparison of changes in ΔT_m_1 and ΔT_m_3 for GS-9669 bound NS5B truncation mutants. Although ΔT_m_3 increases, ΔT_m_1 (st dev 0.1°C) decreases with tail truncation, in agreement with destabilization of polymerase.

The melting profile of apo Δ21 NS5B showed an asymmetric peak that can be described by two distinct transitions: a major transition 1 (associated with T_m_1) and a leading shoulder transition 2 with T_m_2 around 2°C lower (Figure 6A). To ensure that observed transitions were indeed related to unfolding of the monomeric polymerase and not to melting transitions of larger, oligomeric species of NS5B, protein was assayed at 3 concentrations (Figure S4). The T_m_s for all transitions were constant over the range tested. Further analysis by analytical ultracentrifugation of Δ21 and Δ47 showed that both polymerase constructs are predominantly monomeric in a similar concentration range (>95% monomer, data not shown).

When saturating concentrations of representative NNIs were added, the melting profiles of NS5B changed. In general, the measured T_m_ of transitions 1 and 2 were higher in the presence of inhibitors, indicating an increase in protein stability due to the contribution from the free energy of small molecule binding as was observed in the case of thumb site II inhibitors by DSF. The only exception noted was for NS5B bound to a thumb site I ligand. In this case T_m_1 was lowered by around 2°C and T_m_2 was no longer observed (Figure 6C). However, we do not view these results as inconsistent with the SPR binding data reported in Table 1; the phenomenon of a downshift in T_m_ upon compound binding to unfolded/destabilized protein is well established [37], [41], [42], and a T_m_ downshift for thumb site I inhibitors has been reported previously [43], [44]. Moreover, the destabilization of NS5B by thumb site I inhibitors is consistent with the mode of inhibition for this class of NNIs, whereby the polymerase is trapped in a less stable conformation via disruption of the Λ1 finger loop contact with the thumb domain by compound binding at this interface [25], [38].

Transitions observed for Δ21 in the presence of the palm site II inhibitor (Figure 6B) and the palm site I inhibitor (data not shown) mirror the melting profile characteristic of the protein without ligands bound but with the midpoint of unfolding for bot transitions markedly elevated (by around 10°C), suggesting that the polymerase binds these inhibitors without significant changes in conformation. This observation is consistent with the location of the overlapping palm site I and palm site II pockets at the interface of the palm, finger, and thumb domains. Binding of both palm site inhibitors would be expected to stabilize a closed, inactive conformation of the polymerase, consistent with enzymatic and crystallographic studies on these two allosteric sites [20], [45], [46], [47].

The melting profile of Δ21 in the presence of the thumb site II inhibitor GS-9669 is clearly different from those seen with the other NNIs (Figure 6D), and displays three transitions that are strictly related to compound binding as indicated by a dependence of T_m_ upshift on inhibitor concentration (SI and Figure S5). Interestingly, the third transition, with T_m_ slightly higher than T_m_1, is also observed in the presence of the thumb site I inhibitor (Figure 6B–D). The upshift of T_m_s for Δ21 in complex with GS-9669 indicates that overall polymerase is stabilized by binding at thumb site II, as in the case of the palm site I and II binders but in contrast to the decrease in stability observed with thumb site I inhibitors. The appearance of the third transition with T_m_ higher than T_m_1 may be a direct consequence of interaction with the thumb domain, as it is visible only for protein bound to thumb site I and II inhibitors. It is tempting to conclude that the new transition is a manifestation of an independent melting of the thumb domain, but such an assignment remains inferentially based upon this data. DSC, therefore, reveals very different thermal unfolding profiles for NS5B polymerase in the presence of thumb site II as compared to thumb site I inhibitors, consistent with their distinct mechanisms of action.

### Effect of C-terminal truncations on NS5B melting profile in the presence of GS-9669

Analogous to Δ21, the C-terminal truncation constructs Δ39 and Δ47 in the presence of thumb site II inhibitor GS-9669 (Figure 6E–F) display a third transition, with comparable T_m_3. However, the upshifts in T_m_1 and T_m_2 decrease with each successive truncation and is negligible in Δ47 compared to apo protein (Figure 6F–G). Thus, despite the consistent presence of transition 3 for all truncated constructs that stems from GS-9669 binding to the thumb domain, the stabilization that in Δ21 propagated to the entire polymerase is now decoupled from the rest of the protein, implying that critical communication between domains has been disrupted by removal of the C-terminal residues. This is consistent with the hypothesized mechanism of inhibition in which inhibitor binding to the polymerase results in stabilization of the closed conformation (similar to the crystallographic structure) by promoting C-terminal tail contacts with the β-loop, finger and palm domains. Although key regulatory elements of NS5B – the β-loop and the C-terminal tail – do not participate directly in thumb site II inhibitors binding, they play a critical role in exerting the inhibition of enzymatic activity by this class of NNIs. Consequently antiviral resistance could arise both from mutations in the inhibitor binding site and in the key regulatory elements. Mutations of amino acids residues lining the thumb site II pocket (M423T, M423I, M423V, I482L, R422K and L419M) have been generated in replicon-based selection experiments with various thumb site II [27], [48]. Interestingly, an additional cluster of mutations (K441R, A442T and C445F/Y) have also been identified for pyranoindole thumb site II inhibitors [13]. These mutations reside in β strand 7 which forms a part of the β-loop. This observation reemphasizes the importance of β-loop structural motif for allosteric inhibition of NS5B polymerase activity by thumb site II NNIs.

## Conclusion

Several recent studies attempting to explain the mode of action of thumb site II inhibitors have been published [15], [38], [49]. However, while most implicate interference with conformational changes of the enzyme necessary for RdRp activity, none reveal details of the structural dynamics involved. Here we demonstrate that thumb site II inhibitors exhibit a mechanism that is distinct from other classes of NNIs. Despite the ability of thumb site II inhibitors to bind and stabilize all mutant constructs of the polymerase, their ability to inhibit RdRp activity vanishes when the interactions between key structural motifs of NS5B - the β-loop and the C-terminal tail - are weakened or removed. Removal of the β-loop results in a constitutively active enzyme, consistent with a switch to an artificially open polymerase conformation that thumb site II inhibitors can no longer inhibit. This is also consistent with our model of NS5B bound to RNA substrate in elongation mode in which the β-loop is displaced and no longer interacting with the C-terminal tail, allowing the RNA product to exit. A mechanism for the inhibition of NS5B RdRp activity by GS-9669 emerges in which binding at the thumb site II pocket, remote from the active site, locks the entire NS5B polymerase in an enzymatically inactive closed conformation. The stabilization of the closed conformation is a consequence of the communication between the binding pocket on the thumb domain and other domains of polymerase that is mediated by the interactions between two key regulatory elements the β-loop and the C-terminal tail.

## Supporting Information

Figure S1 **Inhibitory potency of GS-9669 towards NS5B Δ55 is significantly decreased in comparison to activity against Δ21.** Overlays of representative dose response curves for selected inhibitors and two NS5B constructs - Δ21 and Δ55 - are shown for: (**A**) 3′dCTP nucleotide inhibitor, (**B**) thumb site II inhibitor GS-9669, (**C**) thumb site I-A inhibitor and (**D**) palm site II inhibitor HCV-796. On average a 89-fold increase in IC_50_ is observed for GS-9669 and Δ55 in comparison to potency towards Δ21, while the active site inhibitor and thumb site I-A inhibitor are unaffected by removal of C-terminal residues (around 1.4 and 0.8-fold change in IC_50_, respectively) and the potency of palm site II inhibitor against Δ55 is decreased on average by 44-fold in comparison to Δ21.
(TIF)

Figure S2 **SPR sensorgrams for binding of NNIs to Δ21, Δ55 & Δ21-Δ8 NS5B constructs.** Kinetic traces obtained for respective NS5B constructs (in columns) binding to various allosteric inhibitors (dilution series, in rows) are shown. (**A**) Thumb site I-B (inhibitor concentration used were 300, 100, 33.3 and 11.1 nM for Δ21 and Δ21-Δ8; or 300, 100, 33.3, 11.1 and 3.7 nM for Δ55); (**B**) Thumb site II - GS-9669 (100, 33.3, 11.1 and 3.7 nM); (**C**) Thumb site II - Lomibuvir (300, 100, 33.3, 11.1 and 3.7 nM); (**D**) Thumb site II - Filibuvir (4000, 1000, 250, 62.5 and 15.6 nM); (**E**) Palm site I inhibitor A-837093 (Δ21 - 100, 33, 11 and 3.7 nM; Δ55 – 100, 33, 11, 3.7 and 1.2 nM; Δ21-Δ8 – 10000, 5000, 2500, 1250 and 625 nM); (**F**) Palm site II - HCV-796 (2000, 1000, 500, 250 and 125 nM). All data sets with exception of data collected for palm site I inhibitor A-837093 on Δ21-Δ8 (E) and palm site II - HCV-796 on Δ55 and Δ21-Δ8 (F) were analyzed using a simple 1 1 kinetic binding model with ProteOn software (solid lines represent the best fit). Association (k_a_) and dissociation (k_d_) rate constants and equilibrium dissociation constant (K_D_) obtained for each fit are provided in Table S1 in File S1.
(TIF)

Figure S3 **Unfolding profiles and thermal unfolding midpoints obtained from DSF and DSC techniques are comparable.** (**A**) DSC thermal unfolding transitions obtained for apo Δ21, Δ39, and Δ47 NS5B truncation mutants (red, green, and blue curves, respectively). DSC unfolding profiles are similar to melting curves obtained by DSF (Figure 4). (**B**) Correlation between T_m_ determined for apo Δ21, Δ39, and Δ47 by DSC and DSF.
(TIF)

Figure S4 **T_m_s for melting transitions of NS5B are independent on protein concentration.** DSC experiments were performed for 2.5, 5, and 7.5 µM of NS5B Δ21 (blue, green, and red tracers, respectively). (**A**) Overlay of raw Cp data. (**B**) The best fit of data in (A) was obtained using a non-two-state unfolding model with two transitions: a major transition 1 (Peak 1, solid line) and the leading shoulder transition 2 (Peak 2, dashed line). For clarity only the overlay of individual first and second transitions fitted for three NS5B Δ21 concentrations is shown. (**C**) Average T_m_ of the first (dark blue bar, T_m_1) and leading shoulder (cyan bar, T_m_2) transitions at each protein concentration (**D**) ΔH for the first (dark blue bar) and second leading shoulder (cyan bar) transitions and total ΔH of unfolding (yellow bar) are shown.
(TIF)

Figure S5 **Unique unfolding transitions observed for NS5B in the presence of GS-9669 depend on the formation of protein-inhibitor complex.** DSC experiments were performed for constant concentration of NS5B (5 µM) and increasing concentrations of GS-9669 (0, 1.25, 2.5, and 25 µM). Traces in graphs are colored as follows: raw DSC Cp (solid black line); overall fit (solid red line); apo major transition with T_m_1° (dashed blue line), apo second transition with T_m_2° (solid cyan line); major transition for NS5B bound to GS-9669 with T_m_1′ (blue line, solid); transition 3 which is visible only in the presence of inhibitor with T_m_3′ (solid green line). (**A**) Unfolding profile for apo Δ21 shown with fit to non-two step unfolding model with two transitions; (**B**)–(**D**) Unfolding transitions for NS5B in the presence of increasing concentrations of GS-9669 (1.25, 2.5, and 25 µM, respectively). Disappearance of the major transition with T_m_1° characteristic of apo Δ21 (dashed blue line) is concomitant with fractional increase of major transition for protein in complex with thumb site II inhibitor (solid blue line, with upshifted T_m_1′) and appearance of third transition with T_m_3 (solid green line) visible only in the presence of thumb site II inhibitor; (**E**) Overlay of the major transitions (with T_m_1) in the unfolding profiles of Δ21 in the absence (blue, dashed) and presence of increasing concentrations (1.25 µM pink, 2.5 µM black, 25 µM blue) of thumb site II inhibitor. Both peaks are anti-correlated with dashed and solid lines indicating transitions for unbound and bound NS5B at each concentration, respectively. (**F**) ΔH of each transition in the melting profile plotted as % area under a peak, showing anti-correlative signature of the major transition for unbound NS5B (blue bar, dashed) and bound in complex with GS-9669 (blue bar, solid) and the appearance of third transition in the presence of thumb site II inhibitor (solid green bar). %ΔH for the second transition is also shown (cyan bar, solid).
(TIF)

File S1 (DOC)
