# Supplementary figures and images for: Structural and Regulatory Elements of HCV NS5B Polymerase – β-Loop and C-Terminal Tail – Are Required for Activity of Allosteric Thumb Site II Inhibitors
Source: PLoS One. 2014 Jan 9;9(1):e84808. doi: 10.1371/journal.pone.0084808 (PMC3886995; doi:10.1371/journal.pone.0084808)

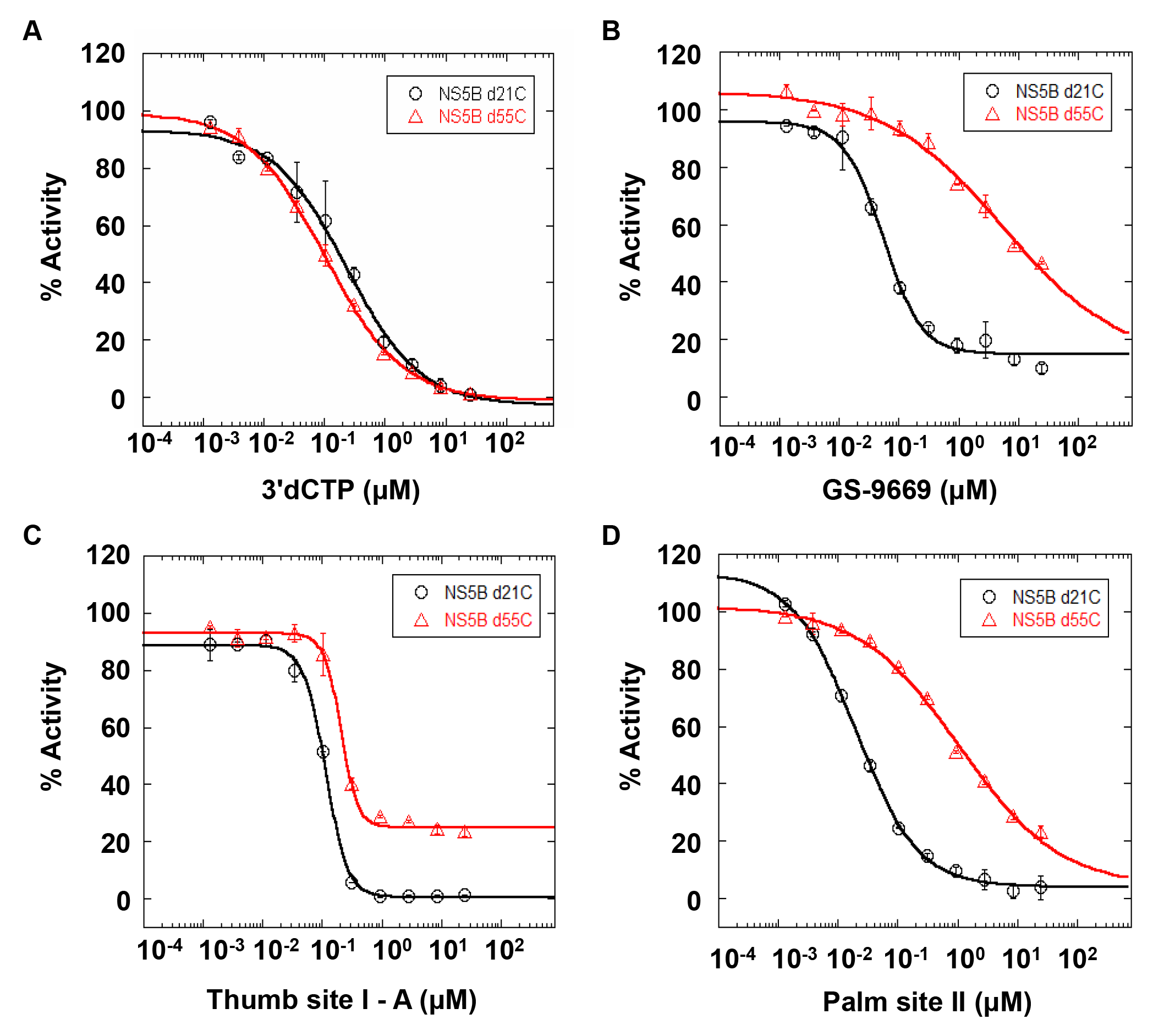

Supplement: Figure S1 — Inhibitory potency of GS-9669 towards NS5B Δ55 is significantly decreased in comparison to activity against Δ21. Overlays of representative dose response curves for selected inhibitors and two NS5B constructs - Δ21 and Δ55 - are shown for: (A) 3′dCTP nucleotide inhibitor, (B) thumb site II inhibitor GS-9669, (C) thumb site I-A inhibitor and (D) palm site II inhibitor HCV-796. On average a 89-fold increase in IC50 is observed for GS-9669 and Δ55 in comparison to potency towards Δ21, while the active site inhibitor and thumb site I-A inhibitor are unaffected by removal of C-terminal residues (around 1.4 and 0.8-fold change in IC50, respectively) and the potency of palm site II inhibitor against Δ55 is decreased on average by 44-fold in comparison to Δ21. (TIF) [file pone.0084808.s001.tif]

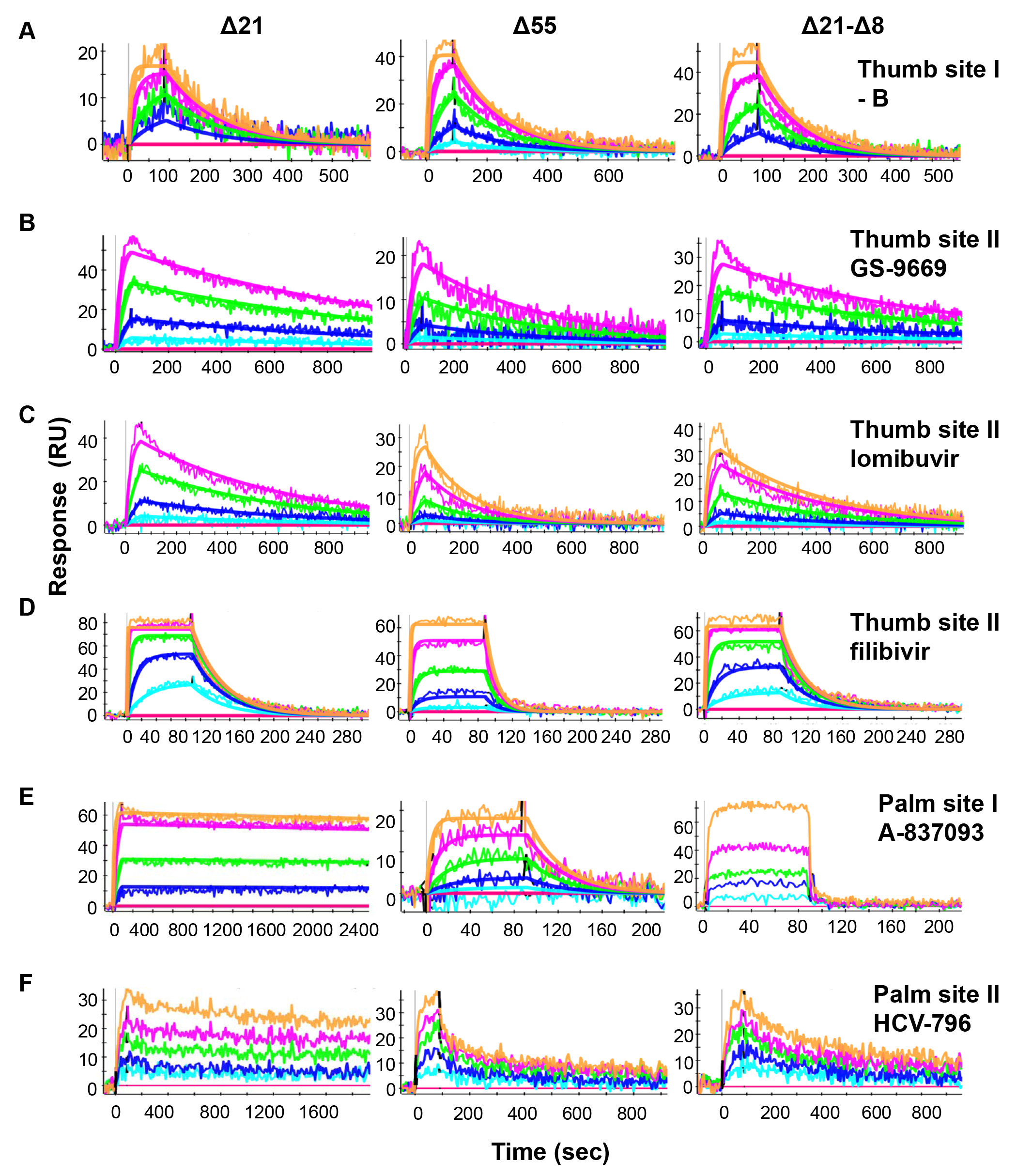

Supplement: Figure S2 — SPR sensorgrams for binding of NNIs to Δ21, Δ55 & Δ21-Δ8 NS5B constructs. Kinetic traces obtained for respective NS5B constructs (in columns) binding to various allosteric inhibitors (dilution series, in rows) are shown. (A) Thumb site I-B (inhibitor concentration used were 300, 100, 33.3 and 11.1 nM for Δ21 and Δ21-Δ8; or 300, 100, 33.3, 11.1 and 3.7 nM for Δ55); (B) Thumb site II - GS-9669 (100, 33.3, 11.1 and 3.7 nM); (C) Thumb site II - Lomibuvir (300, 100, 33.3, 11.1 and 3.7 nM); (D) Thumb site II - Filibuvir (4000, 1000, 250, 62.5 and 15.6 nM); (E) Palm site I inhibitor A-837093 (Δ21 - 100, 33, 11 and 3.7 nM; Δ55 – 100, 33, 11, 3.7 and 1.2 nM; Δ21-Δ8 – 10000, 5000, 2500, 1250 and 625 nM); (F) Palm site II - HCV-796 (2000, 1000, 500, 250 and 125 nM). All data sets with exception of data collected for palm site I inhibitor A-837093 on Δ21-Δ8 (E) and palm site II - HCV-796 on Δ55 and Δ21-Δ8 (F) were analyzed using a simple 1 1 kinetic binding model with ProteOn software (solid lines represent the best fit). Association (ka) and dissociation (kd) rate constants and equilibrium dissociation constant (KD) obtained for each fit are provided in Table S1 in File S1. (TIF) [file pone.0084808.s002.tif]

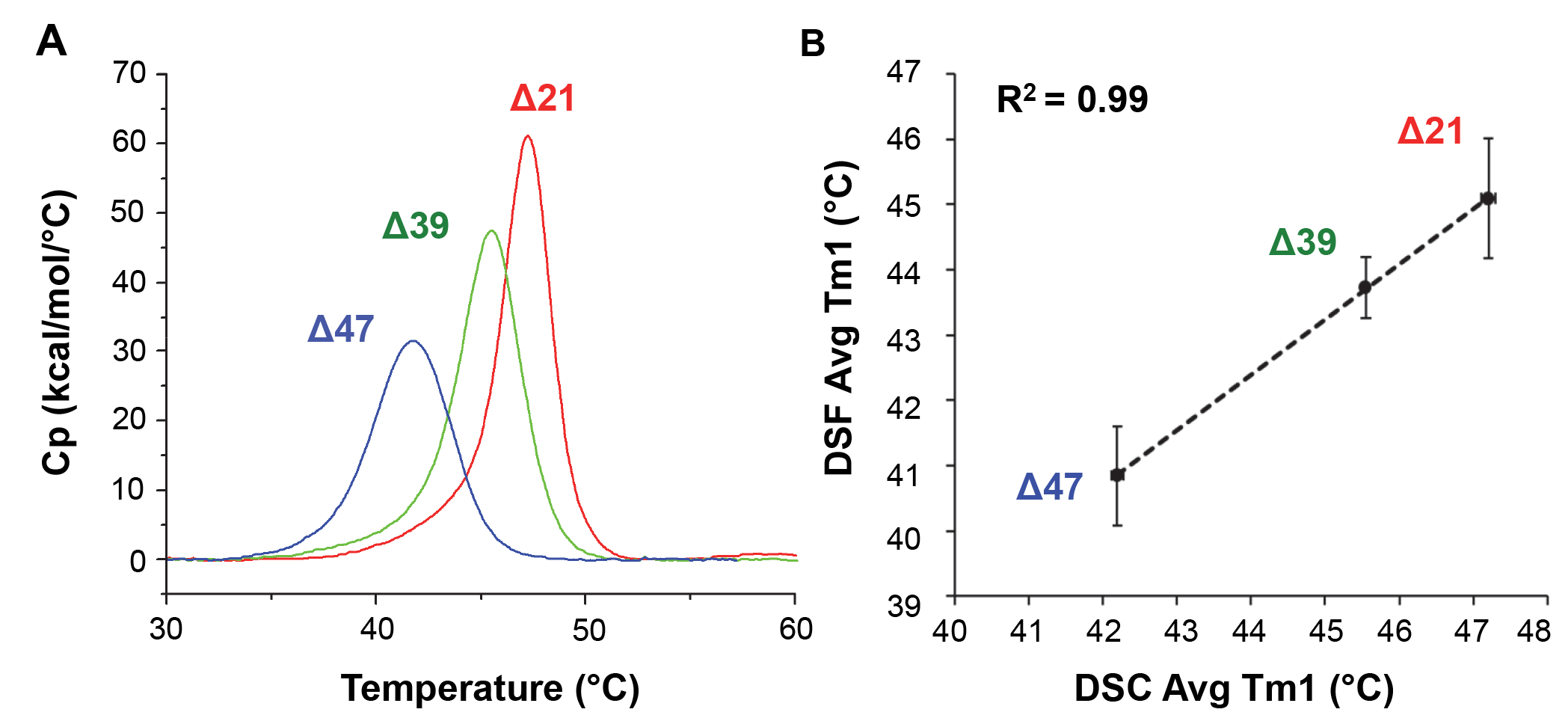

Supplement: Figure S3 — Unfolding profiles and thermal unfolding midpoints obtained from DSF and DSC techniques are comparable. (A) DSC thermal unfolding transitions obtained for apo Δ21, Δ39, and Δ47 NS5B truncation mutants (red, green, and blue curves, respectively). DSC unfolding profiles are similar to melting curves obtained by DSF (Figure 4). (B) Correlation between Tm determined for apo Δ21, Δ39, and Δ47 by DSC and DSF. (TIF) [file pone.0084808.s003.tif]

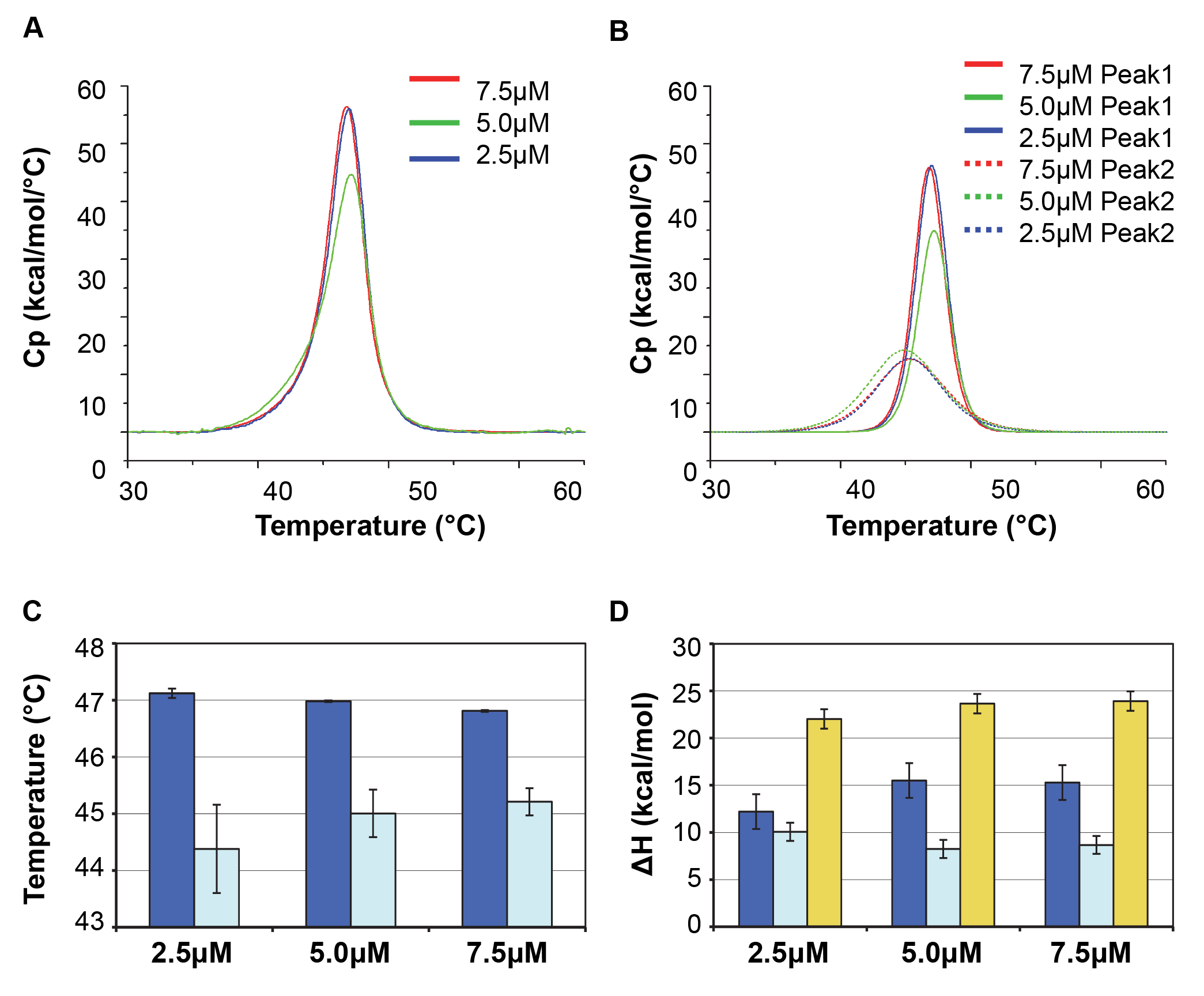

Supplement: Figure S4 — Tms for melting transitions of NS5B are independent on protein concentration. DSC experiments were performed for 2.5, 5, and 7.5 µM of NS5B Δ21 (blue, green, and red tracers, respectively). (A) Overlay of raw Cp data. (B) The best fit of data in (A) was obtained using a non-two-state unfolding model with two transitions: a major transition 1 (Peak 1, solid line) and the leading shoulder transition 2 (Peak 2, dashed line). For clarity only the overlay of individual first and second transitions fitted for three NS5B Δ21 concentrations is shown. (C) Average Tm of the first (dark blue bar, Tm1) and leading shoulder (cyan bar, Tm2) transitions at each protein concentration (D) ΔH for the first (dark blue bar) and second leading shoulder (cyan bar) transitions and total ΔH of unfolding (yellow bar) are shown. (TIF) [file pone.0084808.s004.tif]

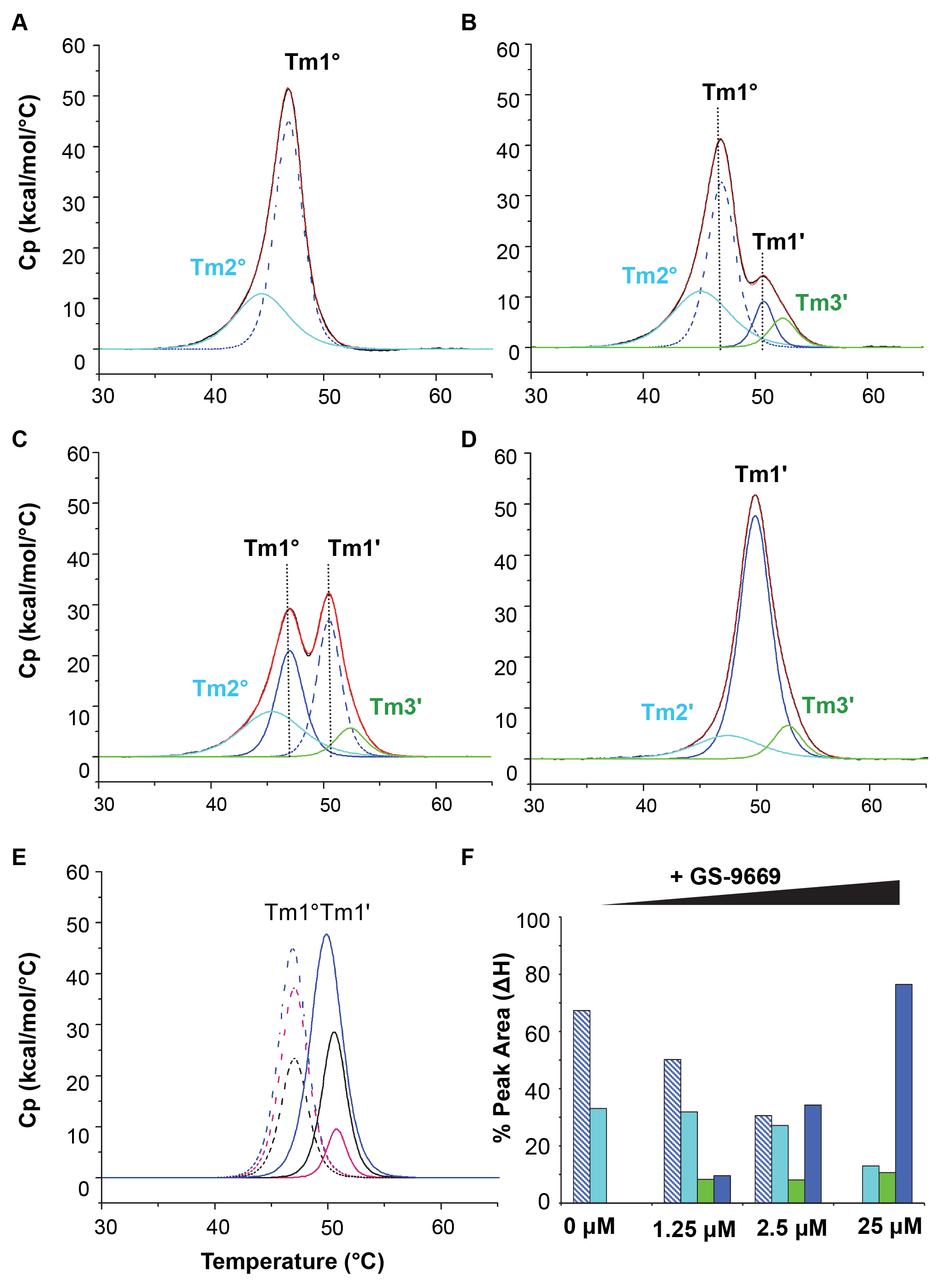

Supplement: Figure S5 — Unique unfolding transitions observed for NS5B in the presence of GS-9669 depend on the formation of protein-inhibitor complex. DSC experiments were performed for constant concentration of NS5B (5 µM) and increasing concentrations of GS-9669 (0, 1.25, 2.5, and 25 µM). Traces in graphs are colored as follows: raw DSC Cp (solid black line); overall fit (solid red line); apo major transition with Tm1° (dashed blue line), apo second transition with Tm2° (solid cyan line); major transition for NS5B bound to GS-9669 with Tm1′ (blue line, solid); transition 3 which is visible only in the presence of inhibitor with Tm3′ (solid green line). (A) Unfolding profile for apo Δ21 shown with fit to non-two step unfolding model with two transitions; (B)–(D) Unfolding transitions for NS5B in the presence of increasing concentrations of GS-9669 (1.25, 2.5, and 25 µM, respectively). Disappearance of the major transition with Tm1° characteristic of apo Δ21 (dashed blue line) is concomitant with fractional increase of major transition for protein in complex with thumb site II inhibitor (solid blue line, with upshifted Tm1′) and appearance of third transition with Tm3 (solid green line) visible only in the presence of thumb site II inhibitor; (E) Overlay of the major transitions (with Tm1) in the unfolding profiles of Δ21 in the absence (blue, dashed) and presence of increasing concentrations (1.25 µM pink, 2.5 µM black, 25 µM blue) of thumb site II inhibitor. Both peaks are anti-correlated with dashed and solid lines indicating transitions for unbound and bound NS5B at each concentration, respectively. (F) ΔH of each transition in the melting profile plotted as % area under a peak, showing anti-correlative signature of the major transition for unbound NS5B (blue bar, dashed) and bound in complex with GS-9669 (blue bar, solid) and the appearance of third transition in the presence of thumb site II inhibitor (solid green bar). %ΔH for the second transition is also shown (cyan bar, solid). [file pone.0084808.s005.tif]
