# Supplementary material for: Structural and Regulatory Elements of HCV NS5B Polymerase – β-Loop and C-Terminal Tail – Are Required for Activity of Allosteric Thumb Site II Inhibitors
Source: PLoS One. 2014 Jan 9;9(1):e84808. doi: 10.1371/journal.pone.0084808 (PMC3886995; doi:10.1371/journal.pone.0084808)
Supplement: File S1 — (DOC) [file pone.0084808.s006.doc]

**SUPPORTING INFORMATION FILE S1:**

**Structural and Regulatory Elements of HCV NS5B Polymerase – β-loop and C-terminal Tail – Are Required for Activity of Allosteric Thumb Site II Inhibitors**

Sarah E. Boyce, Neeraj Tirunagari, Anita Niedziela-Majka, Jason Perry, Melanie Wong, Elaine Kan, Leanna Lagpacan, Ona Barauskas, Magdeleine Hung, Martijn Fenaux, Todd Appleby, William J. Watkins, Uli Schmitz, Roman Sakowicz

**SUPPORTING METHODS**

*HCV NS5B Polymerase Activity Assay.* A 244 nucleotides long heteropolymeric RNA (sshRNA) deprived of secondary structures of the following sequence: 5'-3' (UCAG)_20_(UCCAAG)_14_(UCAG)_20_ was prepared as described previously [1] and was used as a template in the NS5B enzymatic assay. Two-fold dilutions of NS5B starting from 300 nM were prepared in duplicate in 96-well plates. Enzyme was preincubated for 5 minutes at room temperature in a reaction mixture containing 50 mM Tris-HCl, pH 7.5, 10 mM KCl, 1 mM DTT, 0.1 mg/mL BSA, 0.2 unit/µL RNasin Plus RNase Inhibitor (Promega, Madison, WI, cat # N261B), 4 ng/µL sshRNA and 5 mM MgCl_2_. The reaction was initiated by the addition of a mixture containing 2.5 µM ATP, 2.5 µM CTP, 2.5 µM UTP, 1.25 µM GTP, 0.06 µCi/µL of α-^33^P-GTP (3000 mCi/mol, cat # NEG606H, Perkin Elmer, Waltham, MA). Reactions were allowed to proceed for 90 minutes at 30ºC. At various time points, 5 µL of the reaction mixture was spotted on DE81 anion exchange paper (cat # 3658-915, Whatman, UK), which was then washed three times with 125 mM sodium phosphate, pH 9.0, once with water, and once with ethanol. The filter paper was air-dried and exposed to the phosphor imager screen, and the amount of synthesized RNA was quantified using Typhoon Trio Imager and Image Quant TL Software (GE Healthcare, Piscataway, NJ). Reaction rates were calculated by linear regression using GraphPad Prism 5.0 (GraphPad Software, La Jolla, CA).

**SUPPORTING RESULTS**

*Titration of NS5B polymerase with GS-9669 thumb site II inhibitor shows inhibitor concentration dependence on the observed thermal unfolding transitions.* To demonstrate the dependence of the observed transitions on the inhibitor binding site occupancy [2], a titration of NS5B Δ21 in the presence of DMSO alone or 1.25 μM, 2.5 μM and 25 μM of GS-9669 corresponding to approximately 0%, 25%, 50% and 100% of protein-inhibitor complex formation, respectively was performed by DSC (Figure S5A-D). Data were fit to a non-two-state unfolding model with 2, 3 or 4 transitions and apparent T_m_ values for individual transitions were assigned. Analysis with a model invoking four transitions was necessary to obtain best fit (randomly distributed residuals, not shown) of the data collected at the intermediate compound concentrations where both unbound and inhibitor bound protein species are present. For these intermediate concentrations, a peak corresponding to the major transition (transition 1 with T_m_1) for both apo protein and protein-inhibitor complex was observed (Figure S5B and C, dashed and solid blue lines, respectively). Given the presence of transitions corresponding to both bound and unbound NS5B, the following nomenclature is used for each unfolding transition and their corresponding apparent T_m_: T_m_° refers to the midpoint of unfolding transition of a free protein and T_m_' denotes the midpoint of unfolding transition of NS5B in complex with inhibitor. Figure S5E depicts the individual first transitions observed for NS5B in the presence of increasing inhibitor concentrations as NS5B progresses from unbound (with T_m_1°, dashed line) to inhibitor bound species (with T_m_1', solid line). Both T_m_1° and T_m_1' remain constant as the concentration of GS-9669 increases. Additionally, a second transition with T_m_2° observed for apo protein in the DMSO control is also visible in the presence of substoichiometric inhibitor concentration (1.25 μM and 2.5 μM). However, at those intermediate inhibitor concentrations, the second transition for NS5B bound to compound with T_m_2' could not be deconvoluted from the protein unfolding profile (Figure S5B, C). The third transition, characterized by T_m_3', appears only in the presence of thumb site II inhibitor. The %ΔH of unfolding for the third transition does not appear to change significantly with the fraction of complex formed (Figure 5SF, solid green bar). This could be due to the imprecise fitting of additional transitions at the intermediate inhibitor concentrations. However, the %ΔH for the main transition of protein-inhibitor complex (Figure 5SF, solid blue bar) continues to increase, with a corresponding decrease in the apo transition (dashed blue bar) until complete saturation is achieved and no unbound form of the protein remains. The observed anti-correlated switch in the protein unfolding profile which is reliant on inhibitor concentration and therefore concomitant with the transition from the free to the inhibitor-bound polymerase is direct evidence for GS-9669 dependent induction of stabilization of a closed, inactive form of NS5B. This stabilization, as shown by analysis of the conformational and thermal stability of C-terminal truncation constructs of NS5B in complex with GS-9669, is strictly contingent on the proper interaction between β-loop on the thumb domain and C-terminal tail of NS5B, therefore results from a mechanism that is distinct from the mode of action of other NNIs.

**SUPPORTING INFORMATION REFERENCES**

1. Hung M, Gibbs CS, Tsiang M (2002) Biochemical characterization of rhinovirus RNA-dependent RNA polymerase. Antiviral Res 56: 99-114.

2. Shrake A, Ross PD (1990) Ligand-induced Biphasic Protein Denaturation. J Biol Chem 265: 5055-5059.

**SUPPORTING FIGURE LEGENDS**

**Figure S1. Inhibitory potency of GS-9669 towards NS5B Δ55 is significantly decreased in comparison to activity against Δ21.** Overlays of representative dose response curves for selected inhibitors and two NS5B constructs - Δ21 and Δ55 - are shown for: (**A**) 3’dCTP nucleotide inhibitor, (**B**) thumb site II inhibitor GS-9669, (**C**) thumb site I-A inhibitor and (**D**) palm site II inhibitor HCV-796. On average a 89-fold increase in IC_50_ is observed for GS-9669 and Δ55 in comparison to potency towards Δ21, while the active site inhibitor and thumb site I-A inhibitor are unaffected by removal of C-terminal residues (around 1.4 and 0.8-fold change in IC_50_, respectively) and the potency of palm site II inhibitor against Δ55 is decreased on average by 44-fold in comparison to Δ21.

**Figure S2. SPR sensorgrams for binding of NNIs to Δ21, Δ55 & Δ21-Δ8 NS5B constructs.** Kinetic traces obtained for respective NS5B constructs (in columns) binding to various allosteric inhibitors (dilution series, in rows) are shown. (**A**) Thumb site I-B (inhibitor concentration used were 300, 100, 33.3 and 11.1 nM for Δ21 and Δ21-Δ8; or 300, 100, 33.3, 11.1 and 3.7 nM for Δ55); (**B**) Thumb site II - GS-9669 (100, 33.3, 11.1 and 3.7 nM); (**C**) Thumb site II - Lomibuvir (300, 100, 33.3, 11.1 and 3.7 nM); (**D**) Thumb site II - Filibuvir (4000, 1000, 250, 62.5 and 15.6 nM); (**E**) Palm site I inhibitor A-837093 (Δ21 - 100, 33, 11 and 3.7 nM; Δ55 – 100, 33, 11, 3.7 and 1.2 nM; Δ21-Δ8 – 10000, 5000, 2500, 1250 and 625 nM); (**F**) Palm site II - HCV-796 (2000, 1000, 500, 250 and 125 nM). All data sets with exception of data collected for palm site I inhibitor A-837093 on Δ21-Δ8 (E) and palm site II - HCV-796 on Δ55 and Δ21-Δ8 (F) were analyzed using a simple 1:1 kinetic binding model with ProteOn software (solid lines represent the best fit). Association (k_a_) and dissociation (k_d_) rate constants and equilibrium dissociation constant (K_D_) obtained for each fit are provided in Supplementary Table 1.

**Figure S3. Unfolding profiles and thermal unfolding midpoints obtained from DSF and DSC techniques are comparable.** (**A**) DSC thermal unfolding transitions obtained for apo Δ21, Δ39, and Δ47 NS5B truncation mutants (red, green, and blue curves, respectively). DSC unfolding profiles are similar to melting curves obtained by DSF (Figure 4). (**B**) Correlation between T_m_ determined for apo Δ21, Δ39, and Δ47 by DSC and DSF.

**Figure S4. T_m_s for melting transitions of NS5B are independent on protein concentration.** DSC experiments were performed for 2.5, 5, and 7.5 μM of NS5B Δ21 (blue, green, and red tracers, respectively). (**A)** Overlay of raw Cp data. (**B**) The best fit of data in (A) was obtained using a non-two-state unfolding model with two transitions: a major transition 1 (Peak 1, solid line) and the leading shoulder transition 2 (Peak 2, dashed line). For clarity only the overlay of individual first and second transitions fitted for three NS5B Δ21 concentrations is shown. (**C**) Average T_m_ of the first (dark blue bar, T_m_1) and leading shoulder (cyan bar, T_m_2) transitions at each protein concentration (**D**) ΔH for the first (dark blue bar) and second leading shoulder (cyan bar) transitions and total ΔH of unfolding (yellow bar) are shown.

**Figure S5. Unique unfolding transitions observed for NS5B in the presence of GS-9669 depend on the formation of protein-inhibitor complex.** DSC experiments were performed for constant concentration of NS5B (5 μM) and increasing concentrations of GS-9669 (0, 1.25, 2.5, and 25 μM). Traces in graphs are colored as follows: raw DSC Cp (solid black line); overall fit (solid red line); apo major transition with T_m_1° (dashed blue line), apo second transition with T_m_2° (solid cyan line); major transition for NS5B bound to GS-9669 with T_m_1' (blue line, solid); transition 3 which is visible only in the presence of inhibitor with T_m_3' (solid green line). (**A**) Unfolding profile for apo Δ21 shown with fit to non-two step unfolding model with two transitions; (**B**) - (**D**) Unfolding transitions for NS5B in the presence of increasing concentrations of GS-9669 (1.25, 2.5, and 25 μM, respectively). Disappearance of the major transition with T_m_1° characteristic of apo Δ21 (dashed blue line) is concomitant with fractional increase of major transition for protein in complex with thumb site II inhibitor (solid blue line, with upshifted T_m_1') and appearance of third transition with T_m_3 (solid green line) visible only in the presence of thumb site II inhibitor; (**E**) Overlay of the major transitions (with T_m_1) in the unfolding profiles of Δ21 in the absence (blue, dashed) and presence of increasing concentrations (1.25 μM pink, 2.5 μM black, 25 μM blue) of thumb site II inhibitor. Both peaks are anti-correlated with dashed and solid lines indicating transitions for unbound and bound NS5B at each concentration, respectively. (**F**) ΔH of each transition in the melting profile plotted as % area under a peak, showing anti-correlative signature of the major transition for unbound NS5B (blue bar, dashed) and bound in complex with GS-9669 (blue bar, solid) and the appearance of third transition in the presence of thumb site II inhibitor (solid green bar). %ΔH for the second transition is also shown (cyan bar, solid).

**SUPPORTING TABLE**

**Table S1. Kinetic and thermodynamic parameters for binding of selected NNIs to Δ21, Δ55 and Δ21-Δ8 NS5B constructs.**

| **Compound** | **Construct** | **k_a_^a^ ave (1/Ms)** | **k_d_^a^ ave (1/s)** | **K_D_^a^ ave (nM)** | **K_D_^b^ foldshift** |
| --- | --- | --- | --- | --- | --- |
| Thumb site I - B | Δ21 | 5.69E+05 | 5.86E-03 | 12 ± 6 | 1.0 |
|  | Δ55 | 3.91E+05 | 4.43E-03 | 12 ± 3 | 1.0 |
|  | Δ21-Δ8 | 4.31E+05 | 7.88E-03 | 19 ± 6 | 1.6 |
| GS-9669 | Δ21 | 5.37E+05 | 8.34E-04 | 1.55 ± 0.09 | 1.0 |
|  | Δ55 | 2.67E+05 | 1.95E-03 | 9 ± 4 | 5.5 |
|  | Δ21-Δ8 | 4.06E+05 | 1.07E-03 | 2.7 ± 0.3 | 1.7 |
| Lomibuvir | Δ21 | 5.02E+05 | 1.65E-03 | 3.3 ± 0.4 | 1.0 |
|  | Δ55 | 2.17E+05 | 6.28E-03 | 30 ± 9 | 9.0 |
|  | Δ21-Δ8 | 3.56E+05 | 2.29E-03 | 7 ± 2 | 2.0 |
| Filibuvir | Δ21 | 7.41E+05 | 2.74E-02 | 38 ± 13 | 1.0 |
|  | Δ55 | 2.21E+05 | 7.81E-02 | 356 ± 73 | 9.3 |
|  | Δ21-Δ8 | 4.23E+05 | 3.70E-02 | 92 ± 34 | 2.4 |
| A-837093 | Δ21 | 7.67E+05 | 2.71E-05 | 0.04 ± 0.02 | 1.0 |
|  | Δ55 | 1.98E+06 | 3.08E-02 | 17 ± 7 | 432.7 |
|  | Δ21-Δ8 | n/a^#^ | n/a^#^ | n/a^#^ | n/a^#^ |
| HCV-796 | Δ21 | 1.14E+04 | 4.25E-05 | 4 ± 2 | 1.0 |
|  | Δ55 | n/a* | n/a* | n/a* | n/a* |
|  | Δ21-Δ8 | n/a* | n/a* | n/a* | n/a* |

^a^ Association (k_a_) and dissociation (k_d_) rate constants and equilibrium dissociation constants (K_D_) for binding of inhibitors to NS5B constructs were obtained by fitting the sensorgrams (Figure S2) to a single site binding model.

^b^ K_D_ fold shift for binding of particular inhibitor to truncated constructs of NS5B was calculated relative to the K_D_ value measured for Δ21.

^#^ K_D_ values could not be obtained due to super-stoichiometric binding observed for palm site I inhibitor A-837093 and Δ21-Δ8 at high concentrations of compound required to obtain signal, n/a - not applicable.

* K_D_ values could not be obtained due to complex binding kinetics observed for palm site II inhibitor HCV-796 binding to Δ55 and Δ21-Δ8 and numerical values have been replaced by n/a (not applicable)
